# Factors Influencing Anxiety and Depression Symptoms in Patients With Acute Ischaemic Stroke and the Development and Validation of a Risk Prediction Model

**DOI:** 10.62641/aep.v54i3.2226

**Published:** 2026-06-15

**Authors:** Ruorui Yang, Xiaoying Meng, Jingjing Feng, Qiong Zhang

**Affiliations:** ^1^Department of Neurology, Anhui Provincial Corps Hospital of Chinese People’s Armed Police Force, 230001 Hefei, Anhui, China

**Keywords:** acute cerebral infarction, anxiety, depression, influencing factors, predictive model

## Abstract

**Background:**

To analyse the factors influencing anxiety and depression symptoms in patients with acute ischaemic stroke (AIS) and construct a nomogram model for predicting the risk of developing anxiety and depression.

**Methods:**

A total of 354 patients with AIS admitted to the Anhui Provincial Corps Hospital of Chinese People’s Armed Police Force between May 2023 and April 2025 were selected. They were divided into anxiety and depression (n = 180) and nonanxiety and depression (n = 174) groups on the basis of the presence of anxiety (Hamilton Anxiety Rating Scale ≥ 7 points) and depression (Hamilton Depression Rating Scale ≥ 8 points). Baseline patient data, laboratory indicators and anxiety–depression status were extracted from medical records. Logistic multivariable analysis was employed to identify factors influencing the occurrence of anxiety and depression in patients with AIS, establishing a nomogram prediction model. Internal validation was conducted by using receiver operating characteristic (ROC) and calibration curves and decision curve analysis.

**Results:**

Comparative analysis revealed statistically significant differences between the two patient groups in terms of age, neutrophil count, angiogenin-like protein 4 (ANGPTL4), silencing information regulator protein 1 (SIRT1), Krüppel-like transcription factor 2 (KLF2), retinol-binding protein (RBP), lipoprotein (a) levels and plaque stability (*p* < 0.05). Multivariate logistic regression analysis revealed that age (odds ratio [OR] = 1.311, 95% confidence interval [95%CI]: 1.031–1.667), ANGPTL4 (OR = 0.057, 95%CI: 0.023–0.144), SIRT1 (OR = 0.096, 95%CI: 0.016–0.554), KLF2 (OR = 0.001, 95%CI: 0.000–0.401), RBP (OR = 1.476, 95%CI: 1.068–2.040), lipoprotein (a) (OR = 1.130, 95%CI: 1.024–1.247) and plaque stability (OR = 23.941, 95%CI: 5.178–32.186) were factors influencing anxiety and depression symptoms in patients with AIS (*p* < 0.05). The established regression model equation is log(P) = 0.271 × age − 0.044 × ANGPTL4 − 2.348 × SIRT1 − 7.453 × KLF2 + 0.390 × RBP + 0.122 × lipoprotein (a) + 3.176 × plaque stability. The area under the ROC curve was 0.901 (95%CI: 0.867–0.934). Internal validation using the bootstrap method demonstrated high concordance between the predictive and standard model curves.

**Conclusions:**

Age, ANGPTL4, SIRT1, KLF2, RBP, lipoprotein (a) and plaque stability are factors influencing the occurrence of anxiety and depression symptoms in patients with AIS. The nomogram model constructed on the basis of these factors demonstrates predictive validity.

## Introduction

The incidence of stroke in China is rising annually, with acute ischaemic stroke (AIS) accounting for the majority of stroke cases [1]. In addition to physical functional impairments, patients with AIS frequently experience comorbid emotional disorders, such as anxiety and depression, with a prevalence of approximately 35%; these emotional disorders considerably affect the rehabilitation, quality of life and long-term prognosis of this patient population [2]. Therefore, identifying the factors influencing the development of anxiety and depression in patients with AIS and constructing risk prediction models hold considerable clinical importance for the early screening of high-risk populations and the implementation of personalised interventions.

Currently, the clinical identification of poststroke anxiety and depression primarily relies on screening scales administered after symptom onset. However, the pathogenesis of anxiety and depression after stroke is complex, arising from the combined effects of biological, psychological and social factors [3]. In clinical practice, the prevention and management of poststroke mood disorders continue to present numerous challenges. Firstly, their onset is insidious and easily masked by physical symptoms, leading to a high rate of misdiagnosis. Secondly, existing intervention strategies are often initiated only after mood disorders have become apparent, and early warning and risk stratification tools for high-risk populations are lacking. Beyond the psychological effect of the stroke event itself, stroke-related neurobiological alterations, including inflammatory response activation, neuroendocrine dysregulation, structural and functional impairments in specific brain regions and systemic metabolic abnormalities, play a pivotal role in the development of affective disorders. In particular, endothelial dysfunction, dyslipidaemia and chronic inflammation not only contribute to the development and progression of atherosclerosis but may also, by disrupting the blood–brain barrier; activating inflammatory pathways in the central nervous system; and interfering with the function of emotion-regulating brain regions, such as the prefrontal–limbic system, collectively form the pathological basis of poststroke anxiety and depression [4, 5]. Therefore, integrating key biomarkers that reflect these pathways is of great importance for the development of risk prediction models. Risk stratification tools, owing to their visualisation advantages and personalised predictive capabilities, can integrate multiple predictors to display individual disease risk intuitively. They have demonstrated considerable value in prognosis prediction across various conditions [6, 7].

The biochemical parameters selected in this study are all associated with neuroinflammation, oxidative stress, endothelial function and lipid metabolism disorders following AIS. Angiogenin-like protein 4 (ANGPTL4), as a regulator of lipid metabolism, exhibits abnormal expression that may exacerbate atherosclerosis and compromise the integrity of the blood–brain barrier [8]. Silencing information regulator protein 1 (SIRT1) possesses anti-inflammatory and antiapoptotic effects, and its downregulation may activate inflammatory pathways in the central nervous system, a phenomenon that is associated with depressive-like behaviour [9]. Krüppel-like transcription factor 2 (KLF2) influences cerebral microcirculation and neuroimmune dialogue by regulating endothelial function and inhibiting platelet activation [10]. Elevated retinol-binding protein (RBP) levels are associated with oxidative stress and chronic inflammatory states and may contribute to the development of anxiety and depression by inducing a systemic stress response [11]. However, the predictive value of these biochemical parameters for anxiety and depressive symptoms in patients with AIS has not yet been evaluated.

On the basis of the above information, the present study aims to investigate the factors influencing the occurrence of anxiety and depression in patients with AIS and to construct a nomogram prediction model. This endeavour seeks to provide novel insights and quantitative tools for the early screening and precise intervention of anxiety and depression in patients with AIS.

## Materials and Methods

### Research Subjects

A retrospective review was conducted on clinical data from patients with AIS admitted to the Anhui Provincial Corps Hospital of Chinese People’s Armed Police Force between May 2023 and April 2025. A total of 426 patients with AIS admitted to the authors’ hospital underwent eligibility assessment. Of these patients, 72 were excluded for failing to meet the inclusion criteria or for meeting the exclusion criteria. The specific reasons for exclusion were as follows: 15 patients had a history of head trauma or surgery; 21 patients had nonatherosclerotic AIS; eight patients were comatose; 10 patients had severe functional impairment of major organs; seven patients had a history of malignant tumours; six patients had infections or haematological disorders; and five patients had a history of psychiatric disorders. Ultimately, 354 patients were included and completed the study. The sample size was determined in accordance with the principles for estimating sample size in logistic regression models: The model was designed to include seven independent variables representing potential risk factors. By applying the empirical rule that each independent variable requires at least 10–20 positive cases, the required number of positive cases was found to range from 70 to 140. By taking into account the clinical prevalence of anxiety and depressive disorders amongst patients with AIS of approximately 35% (crude) and factoring in a 10% rate of missing clinical data, the required effective sample size was estimated to be between 223 and 445 patients. This study actually included 354 patients who met the requirements for model construction and statistical testing.

Inclusion criteria were as follows: (1) meeting AIS diagnostic criteria [12] confirmed by imaging; (2) time from onset to admission ≤ 72 h; (3) age >18 years; (4) presenting for initial treatment; and (5) complete clinical records. Exclusion criteria included (1) a recent history of head trauma or cranial surgery; (2) AIS caused by other factors, such as atrial fibrillation, trauma, or peripheral vascular disease; (3) patients in a coma; (4) severe dysfunction of major organs; (5) history or medical records of malignant tumours; (6) infectious or haematological diseases; and (7) history of psychiatric disorders, such as depression or anxiety, or currently taking antianxiety or antidepressant medication prior to admission. This study adhered to the Declaration of Helsinki [13] and was approved by the Ethics Committee of the Anhui Provincial Corps Hospital of Chinese People’s Armed Police Force (Ethical Batch Number: WJYY-2025AH-14). Informed consent was obtained from patients.

### Grouping Method

The scale scores recorded in the medical records during a patient’s first 24 h postadmission, when the patient’s consciousness was clear and their condition was stable, were reviewed. Anxiety levels were assessed by using the Hamilton Anxiety Rating Scale (HAMA) [14], which comprises 14 items. High scores on this scale indicate high anxiety severity. The HAMA has a Cronbach’s alpha of 0.832. Depression severity was assessed by using the Hamilton Depression Rating Scale (HAMD) [15], which comprises 17 items and wherein high scores indicate high depression severity. This scale has a Cronbach’s alpha of 0.810. This study allocated patients with HAMA score ≥ 7 and HAMD ≥ 8 to the anxiety and depression group (n = 180), whereas the remaining patients were assigned to the nonanxiety and depression group (n = 174) [16].

### Research Method

Baseline data: Data on all subjects’ gender, age, body mass index (BMI), underlying conditions (hypertension, hyperglycaemia and hyperlipidaemia), smoking history, alcohol consumption history and time from symptom onset to hospital admission were extracted from medical records.

Laboratory parameters: The results of a patient’s fasting blood tests recorded in their medical records following admission were extracted. Fasting blood tests were conducted as follows: A total of 5 mL of fasting blood was collected in the morning after admission. After collection, a patient’s original medical record number was replaced with a random number, and the patient’s plaque stability grouping information was hidden. The inspectors only received samples with random numbers and did not access the patient’ clinical data and grouping results. Serum was separated by using a medical centrifuge (Legend RT, D-37520, Thermo Fisher Scientific, Waltham, MA, USA) at 3500 r/min for 10 min and stored at −70 °C. A fully automated biochemical analyser (BS240, Mindray, Shenzhen, Guangdong, China) was used to measure serum neutrophil count [17], total cholesterol (TC) [18], triacylglycerol (TG) [19], high-density lipoprotein cholesterol (HDL-C) [20] and low-density lipoprotein cholesterol (LDL-C) [21]. Enzyme-linked immunosorbent assay was employed to measure ANGPTL4 [22], SIRT1 [23], KLF2 [24], RBP [25] and lipoprotein (a) [26]. All kits used were purchased from QiYi Biotechnology Co., Ltd. (Shanghai, China), with the following specific models and batch numbers: ANGPTL4 (QY-E10246, Batch Number: 20230415), SIRT1 (QY-E10189, Batch Number: 20230422), KLF2 (QY-E10578, Batch Number: 20230508), RBP (QY-E10367, Batch Number: 20230430) and lipoprotein (a) (QY-E10412, Batch Number: 20230515). All procedures were performed in strict accordance with the manufacturers’ instructions.

Carotid plaque stability: The carotid ultrasound examination report recorded in the patient’s medical records within 24 h of admission was reviewed. All patients underwent carotid ultrasound examination within 24 h of admission by using a colour Doppler ultrasound scanner (EPIQ 7C, Philips Healthcare, Andover, MA, USA). The examination was independently performed by two physicians experienced in vascular ultrasound diagnosis. Carotid thickness was measured at the intima–media thickness 1 cm proximal to the bifurcation of the bilateral common carotid arteries. The average of the measurements from both sides was taken as the final result. Plaques exhibiting a hyperechoic texture with smooth surfaces were classified as stable, whereas those appearing hypoechoic with rough, irregular surfaces were deemed to be unstable [27, 28]. In cases of disagreement between the two physicians, the departmental ultrasound quality control team conducted a review, with the review outcome serving as the final determination.

After the detection was completed, the data manager matched the detection results corresponding to the random number with the original grouping information to establish the final database and thus ensure the absence of subjective interference in detection. The clinical data and laboratory results of all 354 patients included in this study were complete, without missing data.

### Statistical Analysis

All data analyses were performed by using Statistical Product and Service Solutions 27.0 (IBM Corp., Armonk, NY, USA). Continuous variables were expressed as mean ± standard deviation, and comparisons between groups were performed by using independent samples *t*-test. Categorical data were expressed in the form of frequencies and percentages (n [%]), and comparisons between groups were performed by employing the chi-squared test. Logistic multivariate analysis was used to identify the factors influencing the occurrence of anxiety and depression in patients with AIS. Variable selection was performed with forwards stepwise regression, with variables showing *p* < 0.05 in the univariate analysis included as candidate variables in the model. The nomogram prediction model was constructed on the basis of influencing factors, and the bootstrap method (B = 1000) was employed for internal verification. Discrimination by the model was evaluated by utilising the receiver operating characteristic (ROC) curve. The calibration curve and Hosmer–Lemeshow test model were applied to assess accuracy, and decision curve analysis (DCA) was conducted to evaluate the model’s discriminant ability. All statistical tests were conducted through two-tailed tests, and *p* < 0.05 was considered to be statistically significant.

## Results

### Comparison of Baseline Characteristics Between the Two Patient Groups

The results revealed that gender, BMI, underlying diseases (hypertension, hyperglycaemia and hyperlipidaemia), smoking history, drinking history and time from onset to admission did not significantly differ between the two groups (*p* > 0.05). Age showed a statistically significant difference between the two groups (*p* < 0.05) (Table 1).

**Table 1.** **Comparison of general patient characteristics between the two groups**.

Variables		Nonanxiety and depression group (n = 174)	Anxiety and depression group (n = 180)	χ^2^/*t*	*p*
Sex, n (%)				3.090	0.079
	Male	129 (74.14)	118 (65.56)		
	Female	45 (25.86)	62 (34.44)		
Age, year, mean ± SD		58.22 ± 8.46	74.31 ± 9.08	17.236	<0.001
BMI, kg/m^2^, mean ± SD		23.02 ± 1.27	22.78 ± 1.14	1.872	0.062
Underlying diseases, n (%)					
	Hypertension	54 (31.03)	60 (33.33)	0.214	0.644
	Hyperglycaemia	31 (17.82)	35 (19.44)	0.155	0.694
	Hyperlipidaemia	87 (50.00)	92 (51.11)	0.044	0.834
Smoking history, n (%)				0.097	0.756
	Yes	47 (27.01)	46 (25.56)		
	No	127 (72.99)	134 (74.44)		
Drinking history, n (%)				0.899	0.343
	Yes	40 (22.99)	34 (18.89)		
	No	134 (77.01)	146 (81.11)		
Admission time, h, mean ± SD		5.84 ± 2.37	5.76 ± 2.41	0.315	0.753

SD, standard deviation; BMI, body mass index.

### Comparison of Laboratory Parameters, Carotid Intima–Media Thickness and Plaque Stability

The results showed that TC, TG, HDL-C and LDL-C levels did not significantly differ between the two groups (*p* > 0.05). However, neutrophil count, ANGPTL4, SIRT1, KLF2, RBP, lipoprotein (a) level and carotid plaque stability significantly differed between the two groups (*p* < 0.05) (Table 2).

**Table 2.** **Comparison of laboratory parameters, carotid intima–media thickness and plaque stability**.

Variables		Nonanxiety and depression group (n = 174)	Anxiety and depression group (n = 180)	χ^2^/*t*	*p*
Neutrophil count, ×10^9^/L, mean ± SD		4.17 ± 1.12	5.24 ± 1.29	8.322	<0.001
TC, mmol/L, mean ± SD		6.54 ± 1.85	6.72 ± 1.86	0.913	0.362
TG, mmol/L, mean ± SD		1.73 ± 0.47	1.75 ± 0.52	0.379	0.705
HDL-C, mmol/L, mean ± SD		1.12 ± 0.24	1.09 ± 0.27	1.104	0.271
LDL-C, mmol/L, mean ± SD		3.71 ± 0.81	3.82 ± 0.74	1.335	0.183
ANGPTL4, ng/mL, mean ± SD		387.65 ± 31.76	342.72 ± 21.31	15.677	<0.001
SIRT1, ng/mL, mean ± SD		6.89 ± 0.92	4.51 ± 1.75	15.936	<0.001
KLF2, pg/mL, mean ± SD		0.99 ± 0.32	0.54 ± 0.29	13.873	<0.001
RBP, mg/L, mean ± SD		56.91 ± 6.13	72.83 ± 7.54	21.755	<0.001
Lipoprotein (a), mg/L, mean ± SD		202.27 ± 17.94	232.65 ± 19.10	15.414	<0.001
Carotid thickness, mm, mean ± SD		1.42 ± 0.21	1.41 ± 0.23	0.426	0.670
Carotid plaque stability, n (%)				11.517	0.001
	Stable	102 (58.62)	136 (75.56)		
	Unstable	72 (41.38)	44 (24.44)		

SD, standard deviation; TC, total cholesterol; TG, triacylglycerol; HDL-C, high-density lipoprotein cholesterol; LDL-C, low-density lipoprotein cholesterol; ANGPTL4, angiogenin-like protein 4; SIRT1, silencing information regulator protein 1; KLF2, Krüppel-like transcription factor 2; RBP, retinol-binding protein.

### Logistic Multivariate Analysis of Factors Influencing the Development of Anxiety and Depression in Patients With AIS

The factors with *p* < 0.05 from Tables 1 and 2 were incorporated into multivariate logistic regression analysis. The results indicated that age (odds ratio [OR] = 1.311, 95% confidence interval [95%CI]: 1.031–1.667), ANGPTL4 (OR = 0.057, 95%CI: 0.023–0.144), SIRT1 (OR = 0.096, 95%CI: 0.016–0.554), KLF2 (OR = 0.001, 95%CI: 0.000–0.401), RBP (OR = 1.476, 95%CI: 1.068–2.040), lipoprotein (a) (OR = 1.130, 95%CI: 1.024–1.247) and carotid plaque stability (OR = 23.941, 95%CI: 5.178–32.186) were factors influencing the occurrence of anxiety and depression in patients with AIS (all *p* < 0.05) (Table 3).

**Table 3.** **Factors influencing the occurrence of anxiety and depression in patients with AIS**.

Variables	β	SE	Wald	OR (95%CI)	*p*
Age	0.271	0.123	4.879	1.311 (1.031–1.667)	0.025
Neutrophil count	1.445	1.043	1.922	4.243 (0.550–32.748)	0.166
ANGPTL4	–0.044	0.029	2.248	0.057 (0.023–0.144)	0.034
SIRT1	–2.348	0.897	6.853	0.096 (0.016–0.554)	0.010
KLF2	–7.453	3.337	4.989	0.001 (0.000–0.401)	0.012
RBP	0.390	0.165	5.571	1.476 (1.068–2.040)	0.008
Lipoprotein (a)	0.122	0.050	5.959	1.130 (1.024–1.247)	0.019
Carotid plaque stability	3.176	2.501	1.613	23.941 (5.178–32.186)	0.039
Constant	–36.952	39.318	0.883	-	-

AIS, acute ischaemic stroke; SE, standard error; OR, odds ratio; CI, confidence interval; ANGPTL4, angiogenin-like protein 4; SIRT1, silencing information regulator protein 1; KLF2, Krüppel-like transcription factor 2; RBP, retinol-binding protein.

### Nomogram Model of Factors Influencing the Occurrence of Anxiety and Depression in Patients With AIS

**Fig. 1.**
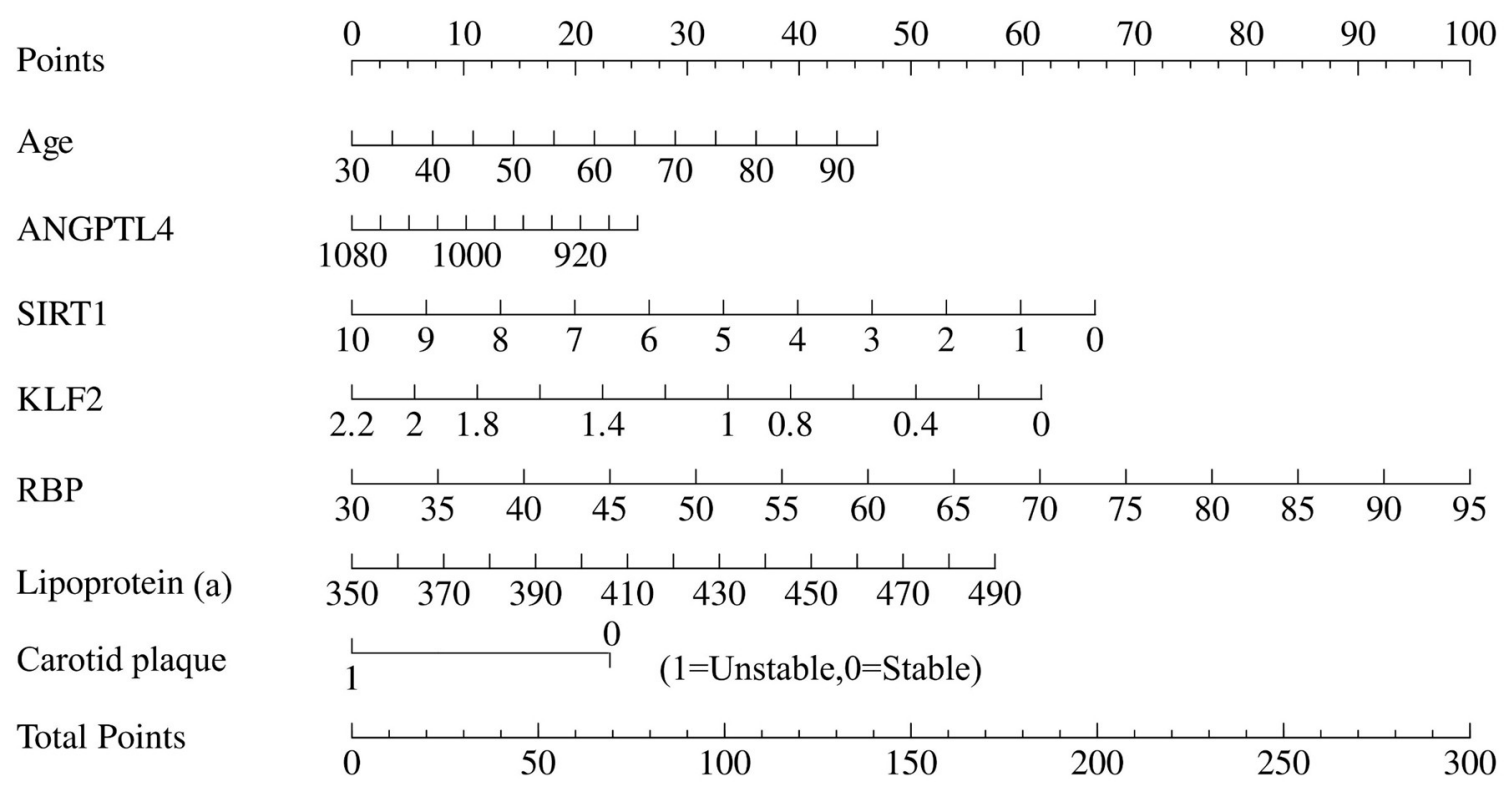
**Nomogram model of factors influencing the occurrence of anxiety and depression in patients with AIS**. ANGPTL4, angiogenin-like protein 4; SIRT1, silencing information regulator protein 1; KLF2, Krüppel-like transcription factor 2; RBP, retinol-binding protein; AIS, acute ischaemic stroke.

The following linear regression model equation for the seven identified influencing factors was established: log(P) = 0.271 × age – 0.044 × ANGPTL4 – 2.348 × SIRT1 – 7.453 × KLF2 + 0.390 × RBP + 0.122 × lipoprotein (a) + 3.176 × carotid plaque stability (Fig. 1).

### ROC Curve Analysis for Regression Model Prediction

**Fig. 2.**
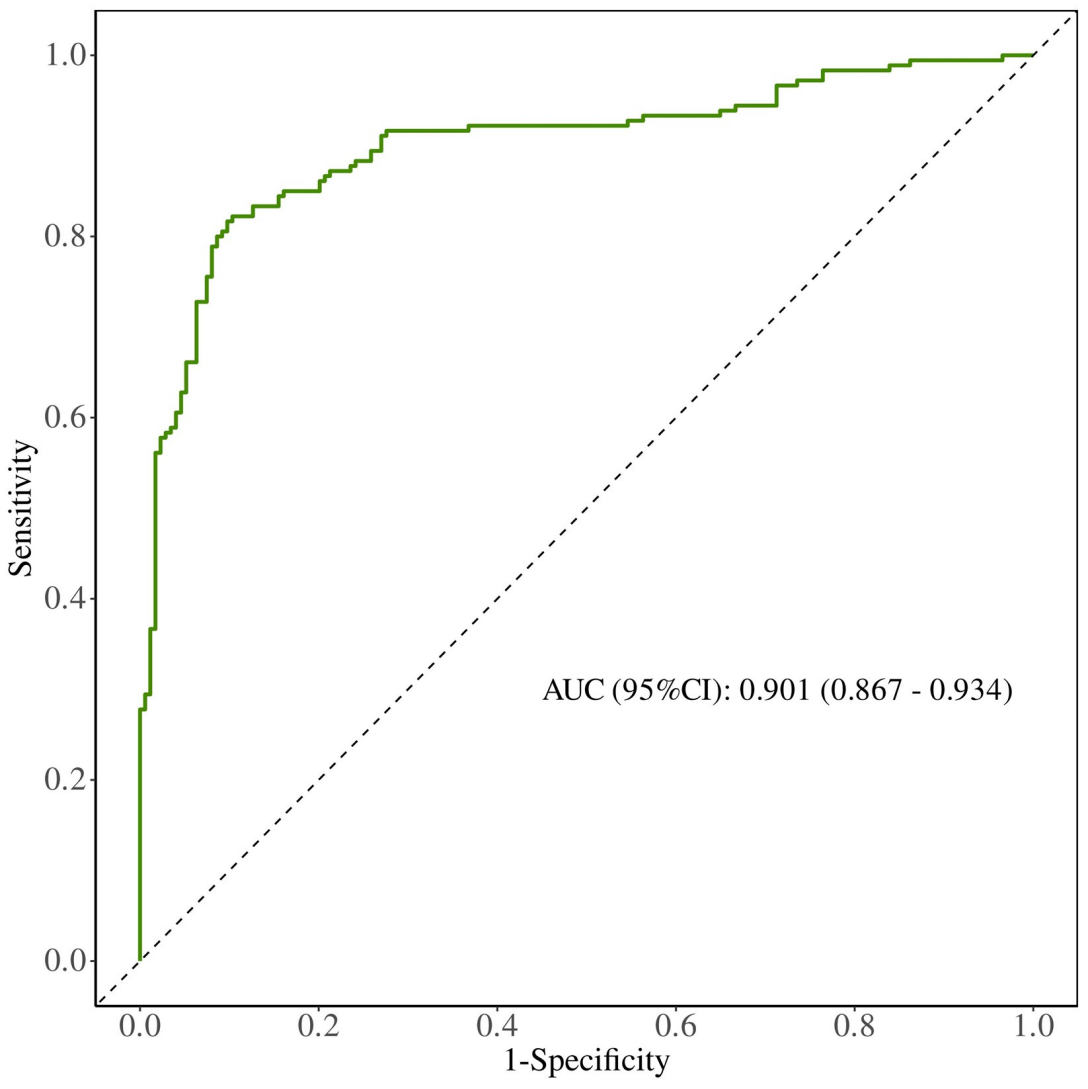
**ROC curve for predicting anxiety and depression in patients with AIS**. AUC, area under the curve; AIS, acute ischaemic stroke.

ROC curve analysis revealed an area under the curve (AUC) of 0.901 (95%CI: 0.867–0.934) for predicting anxiety and depression in patients with AIS, with AUC > 0.7 indicating substantial predictive efficacy (Fig. 2).

### Calibration of Regression Models and DCA Curves

**Fig. 3.**
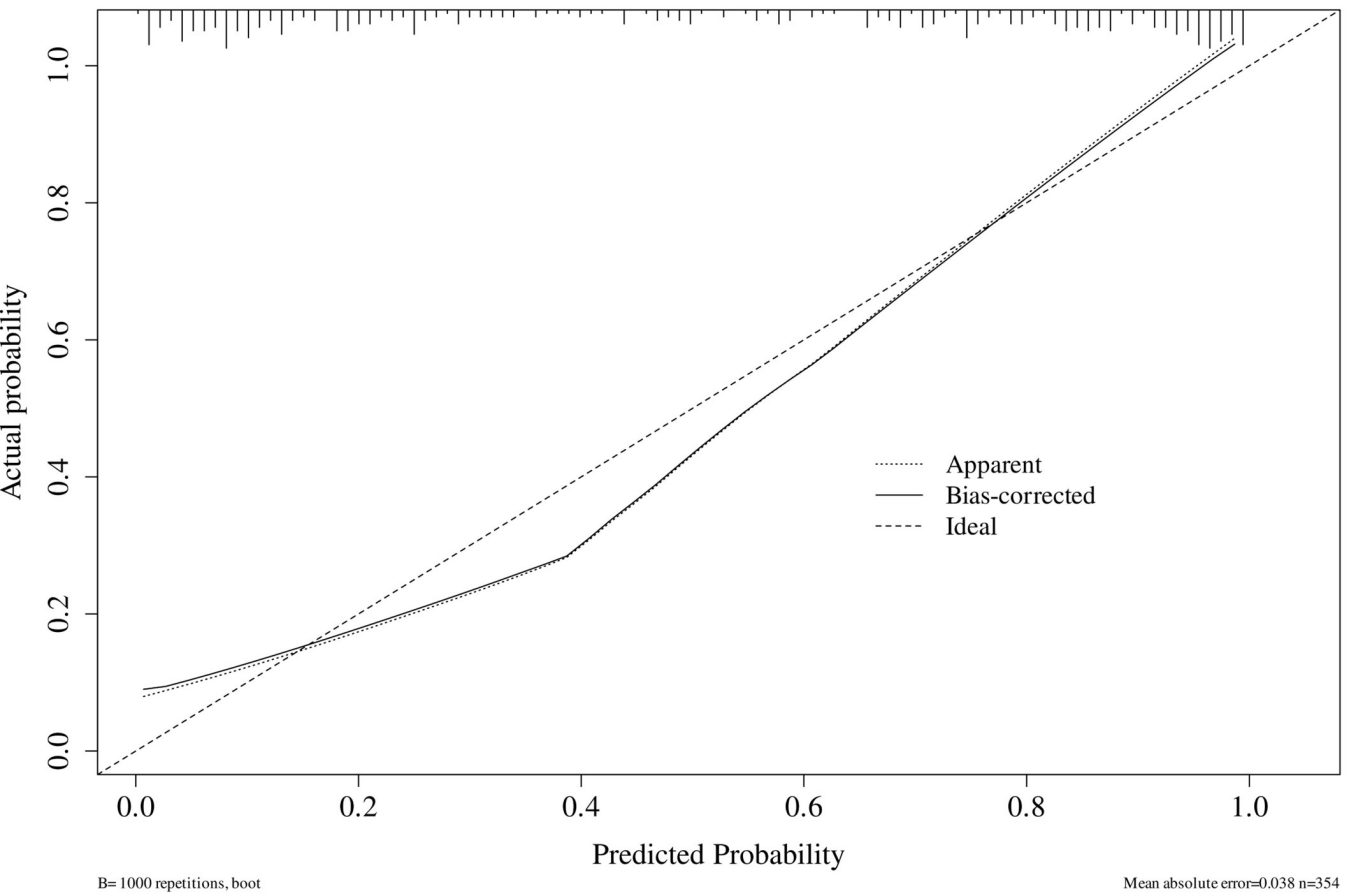
**Calibration curve for predicting anxiety and depression in patients with AIS**. AIS, acute ischaemic stroke.

**Fig. 4.**
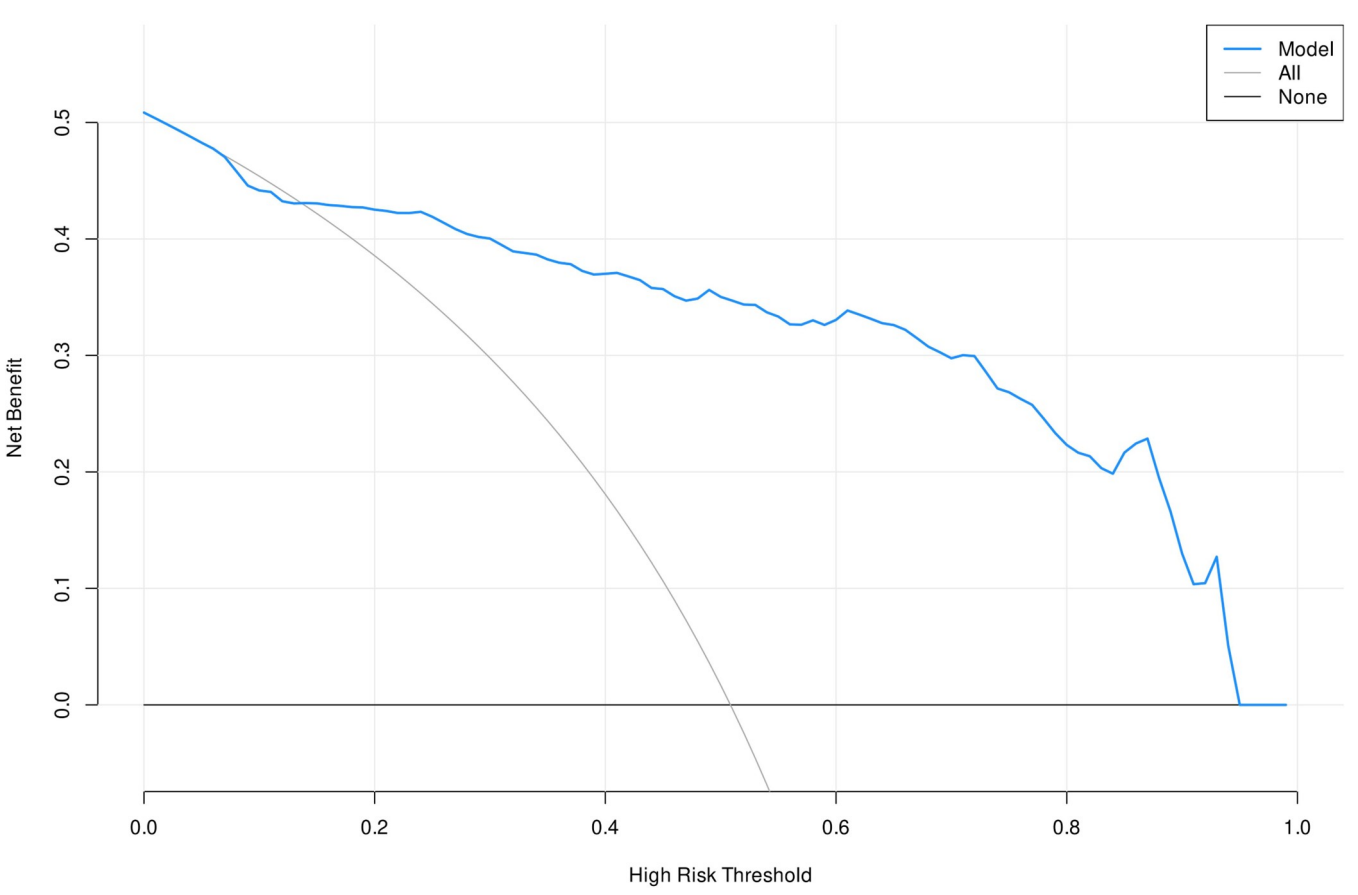
**DCA for predicting anxiety and depression in patients with AIS**. DCA, decision curve analysis; AIS, acute ischaemic stroke.

Internal validation using the bootstrap method demonstrated a high degree of agreement between the predicted and standard model curves (Fig. 3). DCA revealed that the overall trend of the total loss curve for the model decreased as the threshold increased, indicating a consistent response to threshold adjustments during model application (Fig. 4).

## Discussion

AIS occurs when cerebral blood vessels become obstructed or narrow, disrupting blood supply to the brain. This phenomenon leads to oxygen and nutrient deprivation in brain cells, resulting in tissue damage. AIS represents a primary cause of mortality and disability in the Chinese population [29]. Furthermore, it is frequently accompanied with emotional disorders, such as anxiety and depression, markedly impairing the recovery progress, quality of life and long-term prognosis of patients. Therefore, identifying the factors influencing the development of anxiety and depression symptoms in patients with AIS and constructing a risk prediction model holds considerable clinical importance for the early screening of high-risk populations and implementing personalised interventions.

Firstly, this study found that age constitutes a risk factor for anxiety and depression in patients with AIS. With advancing age, patients experience a gradual decline in vascular endothelial function and frequently suffer from multiple chronic underlying conditions, leading to a marked deterioration in physical function and reduced tolerance and coping capacity for illness [30]. In elderly patients, the reduced plasticity of the central nervous system and the impaired functioning of the prefrontal–limbic emotional regulation pathway increase susceptibility to neuroinflammation and neurotransmitter imbalances following a stroke, an effect that in turn can trigger anxiety and depression. Concurrently, elderly patients often possess small social circles and require increased familial support, factors that collectively elevate their risk of developing anxiety and depression [31, 32]. Research by Clayton *et al*. [33] corroborates that elderly patients exhibit a high prevalence of cardiovascular and cerebrovascular diseases. Prolonged illness-related distress readily induces negative emotions, aligning with the present findings. Secondly, ANGPTL4, SIRT1 and KLF2 represent protective factors against anxiety and depression in patients with AIS. ANGPTL4 inhibits lipoprotein lipase activity and regulates triglyceride metabolism [34]. In this study, ANGPTL4 levels in the anxiety and depression group were significantly lower than those in the nonanxiety and depression group (342.72 ± 21.31 pg/mL vs. 387.65 ± 31.76 pg/mL), suggesting that reduced levels may further promote the breakdown of triglycerides and increase the release of free fatty acids and their uptake by macrophages, leading to the formation of foam cells and accelerating the progression of atherosclerosis [35]. Meanwhile, vascular lesions may induce anxiety and depressive moods via neurohumoral regulatory pathways [36]. ANGPTL4 regulates microglial activation by crossing the blood–brain barrier. Its insufficient expression exacerbates poststroke central nervous system inflammation, thereby inducing depression. SIRT1, as a nicotinamide adenine dinucleotide (NAD+)-dependent deacetylase, exerts anti-inflammatory and antioxidant effects. Through deacetylation, it modulates transcription factor activity, thereby suppressing inflammatory responses and oxidative stress [37]. Multivariate analysis in this study revealed that reduced SIRT1 levels constitute a significant risk factor for the development of anxiety and depression in patients with AIS. This result, combined with the significantly lower SIRT1 levels in the anxiety and depression group (4.51 ± 1.75 ng/mL) than in the nonanxiety and depression group (6.89 ± 0.92 ng/mL), suggests that decreased SIRT1 levels may weaken the inhibitory effect of SIRT1 on inflammatory pathways in the central nervous system, thereby contributing to the development of mood disorders. SIRT1 activation can improve synaptic plasticity following a stroke, enhance the function of serotonergic pathways and reduce the risk of anxiety and depression. KLF2 activates the endothelial nitric oxide synthase / nitric oxide (eNOS/NO) pathway to inhibit platelet adhesion, improve the local fibrinolytic environment and downregulate proinflammatory cytokine levels [38]. In this study, KLF2 levels in the anxiety and depression group (0.54 ± 0.29 pg/mL) were significantly lower than those in the nonanxiety and depression group (0.99 ± 0.32 pg/mL). Regression analysis also indicated that reduced KLF2 levels constitute a risk factor for anxiety and depression, suggesting that low KLF2 levels may lead to a diminished ability to inhibit platelet activation and regulate inflammatory responses. When SIRT1 and KLF2 levels decrease, the body’s anti-inflammatory and antioxidant capacities diminish, exacerbating inflammatory responses and oxidative stress in plaques. A deficiency in KLF2 disrupts the vascular endothelial barrier, exacerbates neuroinflammation following cerebral ischaemia and simultaneously affects blood flow and neural function in emotion-related brain regions, thereby becoming a crucial contributing factor to the development of anxiety and depression. Song *et al*. [39] revealed that diminished KLF2 weakens the inhibitory effect of KLF2 on platelet activation, increasing the risk of thrombosis and plaque rupture. This condition further compromises plaque stability, affecting not only vascular lesions but also central nervous system function via neuroinflammatory pathways, thereby heightening susceptibility to anxiety and depression.

This study further identified RBP, lipoprotein (a) and plaque instability as risk factors for anxiety and depression in patients with AIS. Elevated levels of RBP, as a carrier of vitamin A involved in lipid metabolism, are correlated with lipid metabolism abnormalities. This condition can stimulate endogenous oxidative stress, promoting endothelial cell damage and inflammatory responses. Concurrently, physical discomfort arising from vascular lesions and concerns regarding disease prognosis may further precipitate negative emotions. The results of this study indicate that RBP levels in the anxiety and depression group (72.83 ± 7.54 mg/L) were significantly higher than those in the nonanxiety and depression group (56.91 ± 6.13 mg/L). Multivariate analysis revealed that elevated RBP is an independent risk factor for anxiety and depression; it may contribute to the development of mood disorders by activating intravascular inflammatory responses and inducing a chronic stress state. Wang *et al*. [40] indicated that elevated RBP4 levels activate intravascular inflammatory responses, placing the body in a chronic stress state. This phenomenon may constitute a key mechanism for the increased risk of anxiety and depression. Similarly, lipoprotein (a) possesses proinflammatory effects; its elevated levels can increase damage to vascular endothelial cells, promote inflammatory responses and cause further arterial wall injury. This effect complicates patient conditions and rehabilitation, subsequently triggering anxiety and depressive moods [41]. In this study, the level of lipoprotein (a) in the anxiety and depression group (232.65 ± 19.10 mg/L) was significantly higher than that in the nonanxiety and depression group (202.27 ± 17.94 mg/L), and regression analysis showed that elevated levels of lipoprotein (a) were significantly associated with an increased risk of anxiety and depression, further supporting the notion that lipoprotein (a) contributes to the pathophysiological processes of mood disorders via proinflammatory mechanisms. The instability of carotid atherosclerotic plaques signifies that patients face a heightened risk of plaque rupture, thrombus formation and disease recurrence. This concern over disease progression and poor prognosis directly triggers anxiety. Concurrently, vascular stenosis caused by unstable plaques may impair cerebral blood flow [42], thereby affecting the function of brain regions involved in emotional regulation within the central nervous system and inducing depressive mood. When assessing patients with AIS clinically, particular emphasis should be placed on plaque stability indicators to provide targets for early intervention.

In summary, age, ANGPTL4, SIRT1, KLF2, lipoprotein (a) and plaque stability are independent factors influencing the occurrence of anxiety and depression symptoms in patients with AIS. The nomogram model constructed on the basis of these factors demonstrates predictive efficacy. Nevertheless, this study retains the following limitations: Firstly, its sample was drawn from a single hospital. As such, differences in the distribution of patient ages, the spectrum of underlying conditions, the characteristics of vascular lesions and the prevalence of anxiety and depressive disorders, all of which are influenced by regional healthcare resources, clinical practice guidelines and local demographic characteristics, may affect the model’s applicability to different populations, thereby limiting the generalisability of the study’s conclusions. Secondly, potential confounding factors, such as genetic polymorphisms and lifestyle intervention history, were not incorporated, nor were changes in serum biomarker levels dynamically monitored during follow-up, thereby limiting the model’s comprehensiveness. Furthermore, the results of multivariate logistic regression analysis indicate that the confidence interval for the OR of plaque stability is wide, suggesting a degree of uncertainty in the effect estimate for this variable. This situation may be ascribed to the small number of patients with unstable plaques in the anxiety and depression and nonanxiety and depression groups, limiting statistical precision. Plaque stability was assessed solely through carotid ultrasound based on echogenicity and surface morphology and lacked validation by pathological biopsy. Future studies may enhance the accuracy of plaque stability assessment by integrating multimodal imaging or pathological examination. Finally, the nomogram model has not undergone external validation, and its applicability across different healthcare institutions and populations remains unclear. Sample sources must be expanded and external validation must be conducted to improve the model’s clinical applicability and reliability.

## Conclusions

Age, ANGPTL4, SIRT1, KLF2, RBP, lipoprotein (a) and plaque stability constitute risk factors for anxiety and depression symptoms in patients with AIS. The predictive model developed on the basis of these factors demonstrates favourable discriminatory and calibration properties, providing a valuable reference tool for the early clinical identification of high-risk patients. Subsequent studies should further optimise the model through multicentre external validation and explore specific strategies for its integration into clinical workflows.

## Availability of Data and Materials

All experimental data included in this study can be obtained by contacting the corresponding author if needed.
